# Edge-enhanced interaction graph network for protein-ligand binding affinity prediction

**DOI:** 10.1371/journal.pone.0320465

**Published:** 2025-04-08

**Authors:** Dinghai Yang, Linai Kuang, An Hu

**Affiliations:** Xiangtan University, Xiangtan, Hunan, China; National University of Singapore, SINGAPORE

## Abstract

Protein-ligand interactions are crucial in drug discovery. Accurately predicting protein-ligand binding affinity is essential for screening potential drugs. Graph neural networks have proven highly effective in modeling spatial relationships and three-dimensional structures within intermolecular. In this paper, we introduce a graph neural network-based model named EIGN to predict protein-ligand binding affinity. The model consists of three main components: the normalized adaptive encoder, the molecular information propagation module, and the output module. Experimental results indicate that EIGN achieves root mean squared error of 1.126 and Pearson correlation coefficient of 0.861 on CASF-2016. Additionally, our model outperforms state-of-the-art methods on CASF-2013, CASF-2016, and the CSAR-NRC set, showing exceptional accuracy and robust generalization ability. To further validate the effectiveness of EIGN, we conducted several experiments, including ablation studies, feature importance analysis, data similarity analysis, and others, to evaluate its performance and applicability.

## Introduction

Protein-ligand interactions form the foundation of numerous physiological processes within organisms, playing a crucial role in understanding drug mechanisms, discovering novel drugs, and designing targeted therapeutic strategies [1–3]. Accurately predicting Protein-Ligand binding Affinity (PLA) is an essential step in identifying potential drug candidates during the drug development process [4–6]. However, protein-ligand interactions are highly complex and influenced by multiple factors such as molecular structure, interaction sites, and molecular dynamics, which makes affinity prediction more challenging [7,8].

Early methods for affinity prediction primarily relied on traditional experimental techniques, including X-ray crystallography [9] and nuclear magnetic resonance [10]. While these methods deliver fairly accurate affinity data, their high time and cost requirements make them impractical for efficiently analyzing large compound libraries in the early stages of drug screening [11–13]. Consequently, computational methods have increasingly become a research hotspot for affinity prediction [14]. Computer-aided drug design methods, such as molecular docking [15], molecular dynamics simulations [16], and deep learning models [17], provide new insights and tools for rapidly predicting PLA [18,19]. Among these, deep learning models stand out for their remarkable potential, as evidenced by their success in natural language generation [20], image generation [21], and speech detection [22].

In the past, some deep learning methods for affinity prediction have utilized the sequence information of proteins and ligands. These methods feed the sequence data into deep learning models, using sequence-based architectures such as Long Short-Term Memory (LSTM) networks, Convolutional Neural Networks (CNNs), or attention-based Transformer models to predict affinity [23,24]. For example, DeepDTAF [25] employs dilated convolutions to capture long-range interactions and integrates both local features of the protein binding pocket and global features of the entire protein to predict binding affinity. CAPLA [26] leverages cross-attention mechanisms to capture interaction features between protein binding pockets and ligands. By analyzing amino acid sequences or molecular formulas, these models effectively identify specific patterns and local structural features at the sequence level, thereby enabling them to achieve promising performance in predicting binding affinity. However, sequence-based models often struggle with capturing spatial relationships and complex three-dimensional structures. This limitation hampers their ability to capture subtle structural variations and detailed molecular interactions in realistic environments [27].

Graph Neural Networks (GNNs) have emerged in recent years as a powerful tool for modeling the intricate relationships in molecular structures, gaining widespread use in biomolecular studies [28,29]. By representing proteins and ligands as molecular graphs with nodes and edges, GNNs can capture inter-molecular interaction information through graph convolution operations, thereby enabling more precise representations for affinity prediction [30–32]. SIGN [33] introduced a polarity-guided graph attention layer that uses the distance and angle information between atoms. FAST [34] integrates local and global contextual features to predict PLA. IGN [35] represents protein-ligand complexes as ligand graphs, protein graphs, and bimolecular protein-ligand graphs. GIGN [36] applies a heterogeneous interaction layer to unify covalent and non-covalent interactions within the message-passing stage, enabling it to learn node representation more effectively. CurvAGN [37] introduced curvature blocks and adaptively guided neural blocks to encode edge attributes in biomolecular graphs. LGN [38] enhances the capture of local and global features in protein-ligand complexes by incorporating extra ligand feature extraction. PIGNet [39] employs a physics-informed GNN, which performs excellently in scoring and screening tasks. PIGNet2 [40] further enhances model generalizability by expanding training data with a broader range of binding conformations and ligand types. These GNN-based methods perform well in affinity prediction. However, most existing models typically combine inter- and intra-molecular interactions, which may limit their ability to capture local structural details. Although some studies have attempted to handle these two types of interactions separately, the methods remain relatively simplistic, making it challenging for models to extract rich local information and accurately capture complex molecular interactions.

In this paper, we propose a GNN-based method for predicting PLA. The method focuses on refining the modeling of interactions within protein-ligand complexes by utilizing the inter- and intra-molecular message-passing modules. To enhance the representational power of edge features for capturing interaction information between nodes better, we employ an edge update mechanism that integrates node feature information into edge features. This design helps the model leverage edge information to update node features effectively during message passing. We evaluate the performance of the model on the test set and compare it with state-of-the-art methods. The results demonstrate the superior performance of EIGN. Additionally, several experiments were conducted to evaluate the model in a comprehensive manner, such as ablation studies, feature importance analysis, data similarity analysis, and others.

## Materials and methods

### Database

In this study, we utilized the PDBbind v2020 database as the primary data source. PDBbind [41] is a database of high-quality protein-ligand complexes, designed for structural biology and computational chemistry. It is extensively utilized in drug discovery and molecular modeling research [42]. Compiled from the Protein Data Bank, this database provides a comprehensive collection of protein-ligand complexes, offering extensive data support for studying protein-ligand interactions.

The PDBbind database provides structural information of complexes along with their experimentally measured binding affinities (e.g., dissociation constant *K*_*d*_, inhibition constant *K*_*i*_), which form a reliable foundation for building and validating PLA prediction models. PDBbind v2020 includes a greater number of updated complex data compared to earlier versions, significantly enhancing the comprehensiveness and accuracy of the database. Specifically, PDBbind v2020 (N = 19,443) was randomly divided into two subsets: one subset as the model training set (N = 16,954) and the other subset as the validation set (N = 2,000). During the dataset partitioning, we excluded samples that overlapped with the test set as well as samples that could not be processed by RDKit. The test set consisted of CASF-2013 (N = 195) and CASF-2016 (N = 285) from the PDBbind database [43,44].

In addition to the PDBbind database, we also used a portion of the CSAR-NRC set (N = 85) as an additional test set to further evaluate the generalization capability and predictive performance of the model. The CSAR-NRC set is one of the datasets released by the Community Structure-Activity Resource (CSAR) to provide high-quality PLA data for validating and comparing various virtual screening and molecular docking models [45]. This dataset includes a rigorously filtered set of protein-ligand complexes with precisely measured binding affinities, making it one of the standard datasets for assessing affinity prediction models.

### Protein–ligand complex representation

Graph structures are particularly well-suited for representing protein-ligand complexes, as they effectively capture atomic interactions and the topological information within the complex [46]. Interactions between proteins and ligands (such as drugs) occur at the active sites of proteins, which are some depressions and cavities, usually known as binding pockets [47]. In this study, we defined the protein-ligand interaction region using a distance threshold of 5.0 Å, considering only residues within this range around the ligand. This threshold was selected based on previous studies [48], balancing prediction accuracy and computational cost.

To represent these interactions, we constructed two types of graphs: one for inter-molecular interactions and another for intra-molecular interactions. In these graph structures, each node represents an atom, and we used six types of information to construct the features of these nodes. These features were processed through one-hot encoding and transformed into vector representations. The edges between nodes were represented using either Euclidean distance or node degree.

A notable aspect of our approach is the unified representation method for both protein and ligand atoms. We applied the same representation method without additional feature distinctions, ensuring generality and scalability. The specific details of node features are summarized in Table 1.

**Table 1:** Node feature inputs.

Atom Feature	Description
Atom Symbols	The element symbol of the atom, e.g., C, N, O, S, F, etc.
Degree	The number of neighboring atoms bonded to the atom.
Implicit Valence	The number of implicit valence electrons for the atom.
Hybridization	The hybridization type of the atom.
Aromatic	Indicates whether the atom is aromatic.
Total Hydrogens	The number of hydrogen atoms bonded to the atom.

**Fig 1:**
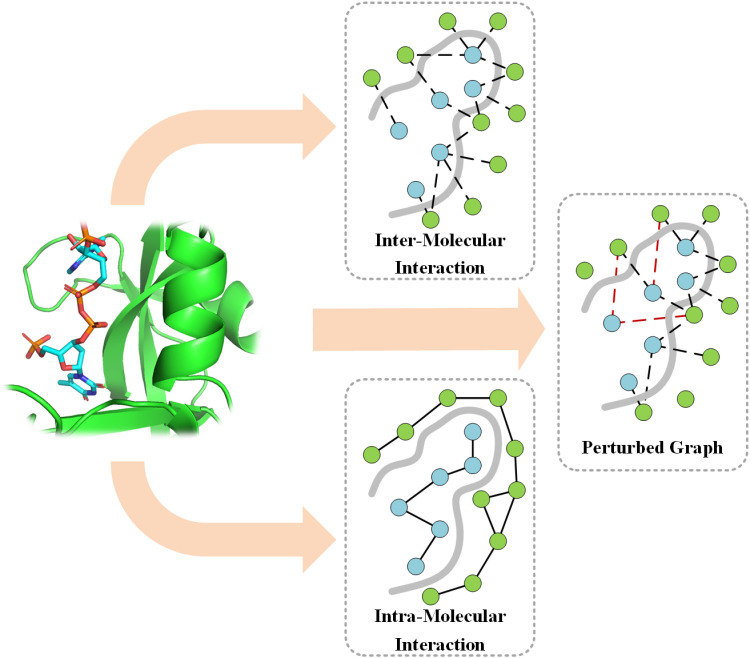
The three types of graphs used in EIGN.

In addition to the mentioned feature processing, we introduced an edge augmentation strategy to better handle intermolecular interactions. Specifically, during graph construction, we randomly delete certain edges to simulate structural noise that may result from docking errors or data quality issues. For instance, we removed edges between nodes at greater distances (e.g., edges exceeding 4 Å). Meanwhile, the edge enhancement strategy randomly adds new edges to enrich the diversity of the graph, allowing the model to contact different graph structures during the learning process. The three types of graphs used in EIGN are illustrated in Fig 1.

### Model architecture

**Fig 2:**
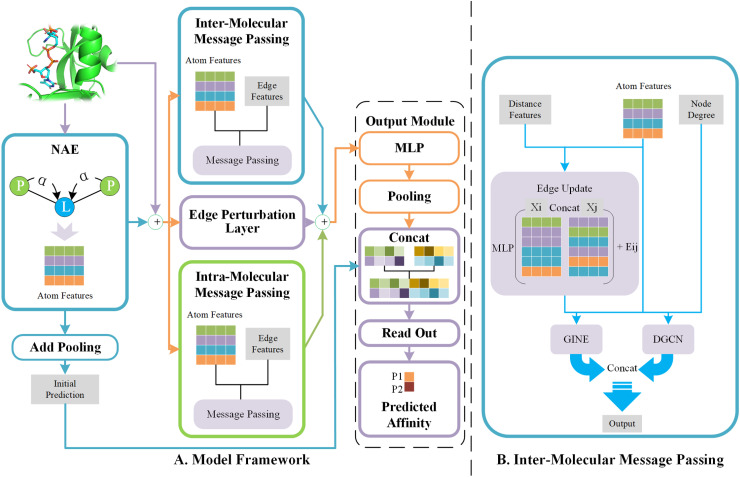
Model architecture. (A) Overall framework of EIGN. (B) Inter-molecular message passing structure.

The framework of the EIGN model is shown in Fig 2A. The input of the model is the protein-ligand complex. We divide the overall model into three modules: Normalized Adaptive Encoder (NAE), the molecular message propagation module, and the output module. The first two modules primarily use GNN to extract and update the feature representations of the nodes. The node features updated by the first two modules are passed to the output Module to generate the final prediction.

#### NAE.

NAE is an encoder module that integrates linear transformations of graph features, a graph propagation mechanism, and global pooling operations. To capture higher-order relationships between nodes, we introduced an enhanced graph propagation mechanism—Approximate Personalized Propagation of Neural Predictions (APPNP) [49]. APPNP employs a “skip connection” gating mechanism that uses the hyperparameter *α* to regulate the weighted combination of initial and propagated features. This mechanism, compared to traditional GNNs, ensures that the influence of the initial features is retained at every layer, thereby effectively mitigating the over-smoothing problem. Consequently, APPNP enables NAE to capture long-range dependencies more effectively and enhances its performance on molecular graph tasks. To ensure numerical stability during the information propagation process, we apply L2 normalization to the node features before propagation. The normalized features are then amplified by a scaling factor, improving their ability to convey information during propagation. These normalized features are passed into the propagation module for further integration of the structural information of the graph. Unlike traditional GNNs, our final step incorporates the node features *X*^′^ after propagation and the graph-level embedding $X_{g}$ obtained through sum pooling, as the result of NAE. The following formula illustrates the execution flow of NAE:


$$\begin{array}{c} H=SILU\left(WX+b\right) \end{array}$$ (1)

$$\begin{array}{c} X^{\prime }=s\frac{H}{∥H{∥}_{2}}\left(1-\alpha \right)+s\frac{H}{∥H{∥}_{2}}\alpha P \end{array}$$ (2)

$$X_{g}=\sum_{v\in G_{i}}X_{v}^{\prime }$$ (3)

where *H* represents the output after feature transformation. *W* is the weight matrix for the linear transformation. *X*^′^ denotes the propagated node features. *s* is the scaling factor, set to 1.8 in this study. *α* represents the propagation rate, set to 0.1. *P* is the normalized adjacency matrix. $X_{g}$ is the graph-level embedding. $G_{i}$ represents the set of all nodes in graph *i*.

#### Molecular message propagation module.

The molecular message propagation module is divided into three parts: inter-molecular message passing, intra-molecular message passing, and the edge perturbation layer. The first two components handle inter-molecular and intra-molecular interactions, while the edge perturbation layer processes the inter-molecular interactions after edge perturbation.

In the inter-molecular message passing module, we designed a mechanism that combines different types of graph convolutional layers to capture the complex structural features within molecular graphs. Fig 2B illustrates the detailed structure of this module. This module updates the node features using both GINE and DGCN, and the final output is obtained by concatenating the node features updated by each method. The GINE layer is an enhanced variant of the Graph Isomorphism Network (GIN) [50]. It allows edge features to be used as input and dynamically integrates them during message passing to improve node feature updates. In this study, edge features processed by the edge update mechanism are fed into the GINE layer to further boost the expressive power of the node features. DGCN module enriches node feature representations with a multi-stage message passing strategy. It first applies a Graph Convolutional Network (GCN) to process the input node features and extract local structural information. Next, the feature update process introduced an edge weighting mechanism based on node degree, which propagates and adjusts node features globally through a custom message passing layer. The resulting features from both parts are concatenated as the output of the DGCN module, hence effectively combining local and global features. The node feature update process in the DGCN module can be described as follows:


$$\begin{array}{c} h^{\left(1\right)}=GCN\left(x\right) \end{array}$$ (4)

$$\begin{array}{c} h^{\left(2\right)}=\underset{j\in N\left(i\right)}{\sum }\left(deg_{i}^{-0.5}\cdot deg_{j}^{-0.5}\cdot x_{j}\right)+b+h^{\left(1\right)} \end{array}$$ (5)

$$\begin{array}{c} out=Linear\left(Concat\left(h^{\left(1\right)},h^{\left(2\right)}\right)\right) \end{array}$$ (6)

where $N\left(i\right)$ represents the set of neighboring nodes of node *i*. $deg_{i}$ is the number of neighbors of node *i*. $x_{j}$ denotes the node features of neighboring node *j*.

The primary function edge update mechanism is to dynamically update the edge features in the graph by combining the feature information of two nodes, enhancing the model’s ability to represent node relationships. By integrating node features and processing them through a Multi-Layer Perceptron (MLP), the mechanism refines the feature representation of each edge, thereby enhancing the ability of the graph structure to capture intricate relationships. This mechanism captures detailed interaction patterns between nodes, offering richer edge information to the GNN, which ultimately improves the effectiveness of graph data modeling. The workflow of the edge update mechanism is illustrated in Fig 2B.

For intra-molecular message passing, the module structure is similar to inter-molecular message passing, but it focuses on the node and edge features within the molecule. Since the internal structure of the molecule contains unique local structural features, intra-molecular message passing helps capture micro-interactions within the molecule by focusing on the local flow of information within the molecular nodes.

The edge perturbation layer addresses inter-molecular interactions influenced by edge perturbations. It aims to enhance the diversity and robustness of the graph structure, enabling the capture of rich inter-node relationships, especially those that may emerge as potential structural information after edge addition or removal. Unlike the previous two sub-modules, this module uses only GIN for graph processing and does not utilize edge feature inputs.

Finally, in the molecular message propagation module, the node features updated through the three sub-modules are combined, and the combined features are used as the final output of the module.

#### Output module.

As shown in Fig 2A, the output Module mainly consists of an MLP and a pooling layer. Its role is to convert node-level features into a global graph-level representation through fully connected layers, pooling, and fusion operations, which are then used for predicting PLA. What makes this module unique is its approach of separately integrating the graph-level embeddings from both the NAE and molecular message propagation module. This enables the model to capture the interactions between local and global features, ultimately improving its ability to model complex structural relationships.

### Implementation details

The model training was conducted on a single GPU, with a batch size of 128 and a dropout rate of 0.1. For optimization, the Adam optimizer was employed, utilizing a learning rate of 5e-4 and a weight decay of 1e-6. The training was conducted for 800 epochs with early stopping implemented, terminating the training process if there was no improvement in performance on the validation set for 100 consecutive epochs. The model used the mean squared error loss function as the objective to evaluate the regression performance.

## Results and discussion

This section comprehensively evaluates the performance of the EIGN model in PLA prediction using two metrics: Root Mean Squared Error (RMSE) and Pearson correlation coefficient (*R*_p_). The experiments show that EIGN outperforms state-of-the-art methods on the PDBbind core set and CSAR-HIQ-set, demonstrating strong generalization ability and prediction accuracy. Ablation studies confirm the effectiveness of key modules within the model, while analyses of feature contribution and similarity-based sample ratios further identify the crucial factors influencing model performance. In addition, we evaluated the performance of the model in virtual screening tasks and its capability to handle unknown target and ligand structures.

### Experiments on the PDBbind core set

We used three datasets to train the model: the refined set of PDBbind v2020 (N = 4473), the general set with refined samples removed (N = 12585), and the complete general set (N = 16954). Each training set was repeated three times, each with a different random seed. The mean and standard deviation of the three model predictions were used as performance indicators. The experimental results are shown in Table 2.

**Table 2:** Experimental results on the PDBbind core set.

Test Set	Training Set Samples	RMSE	*R*_p_
CASF-2013/195	4473	1.470 ± 0.008	0.758 ± 0.004
	12585	1.499 ± 0.037	0.747 ± 0.014
	16954	1.284 ± 0.020	0.826 ± 0.008
CASF-2016/285	4473	1.27 ± 0.009	0.815 ± 0.004
	12585	1.277 ± 0.024	0.813 ± 0.006
	16954	1.126 ± 0.028	0.861 ± 0.010

As shown in Table 2, the model trained on the refined set and the model trained on the general set with the refined samples removed demonstrate similar performance on both test sets. However, the general set contains more data noise, which leads to more fluctuation in the prediction results, as evidenced by the larger standard deviations of RMSE and *R*_p_ shown in Table 2. The complete general set strikes a balance in terms of data scale and diversity, leading to the best generalization ability for the trained model.

**Fig 3:**
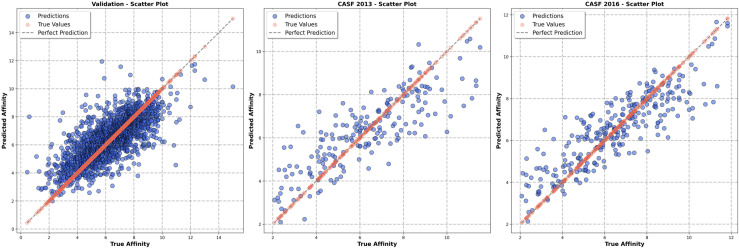
Scatter plots of predicted values (y-axis) versus actual values (x-axis) for EIGN on the validation set, CASF-2013, and CASF-2016.

Fig 3 presents the scatter plots of affinity prediction and actual affinity values for EIGN trained on the PDBbind v2020 general set, evaluated on the validation set, CASF-2013, and CASF-2016. From the scatter plot of the validation set, the model predictions generally align with the actual values. However, in the extreme value regions, the model performs poorly. This issue may arise from the scarcity of extreme samples and the imbalance in the data, which prevents the model from accurately predicting the affinity values for these cases. The predicted and actual values for the two test sets also show a clear linear relationship.

### Comparison with state-of-the-art methods

To verify the superiority of EIGN, we compared its performance against several state-of-the-art methods on CASF-2013 and CASF-2016. To ensure a fair comparison, we limited the evaluation to methods that utilized either the PDBbind v2016 or PDBbind v2020 datasets for training. A total of seven methods were selected for this comparison: OnionNet [51], SIGN [33], FAST [34], IGN [35], GIGN [36], CurvAGN [37], and LGN [38]. Specifically, we reproduced IGN and GIGN and trained them on the same datasets used for EIGN.

Table 3 presents the results of this comparison. The results highlight that our model consistently outperforms the other methods. Regardless of whether it was trained on the PDBbind v2016 or PDBbind v2020 datasets, EIGN demonstrates superior performance and surpasses all other methods in the comparison. It is important to note that although some methods use the same version of the dataset (e.g., PDBbind v2016 or PDBbind v2020), the number of training samples varies. This discrepancy arises from differences in preprocessing and filtering criteria employed in different studies. For instance, the GIGN method excludes samples that cannot be processed using RDKit. In contrast, CurvAGN does not filter any samples. Additionally, the significant difference in the number of samples in CASF-2013 is due to some methods only using the subset of samples that overlap between CASF-2013 and CASF-2016, rather than the complete CASF-2013 sample set.

**Table 3:** Comparison of predicted results between EIGN and state-of-the-art methods on CASF-2013 and CASF-2016.

Model	Training set/Sample quantity	CASF-2013			CASF-2016		
		Sample quantity	RMSE	*R*_p_	Sample quantity	RMSE	*R*_p_
FAST	PDBbind v2016/11717				290	1.308	0.81
OnionNet	PDBbind v2016/11906	108	1.503	0.782	290	1.278	0.816
SIGN	PDBbind v2016/11903	107	1.448	0.79	285	1.272	0.816
CurvAGN	PDBbind v2016/11993				290	1.217	0.831
LGN	PDBbind v2016/11517				285	1.177	0.842
IGN	PDBbind v2020/16954	195	1.44	0.772	285	1.303	0.805
GIGN	PDBbind v2020/16954	195	1.381	0.791	285	1.278	0.81
**EIGN(ours)**	**PDBbind v2016/11816**	**194**	**1.249**	**0.837**	**285**	**1.131**	**0.857**
**EIGN(ours)**	**PDBbind v2020/16954**	**195**	**1.284**	**0.826**	**285**	**1.126**	**0.861**

For the model trained on PDBbind v2016, EIGN outperforms LGN on CASF-2016 with a 4% decrease in RMSE and a 2% increase in *R*_p_. When trained on PDBbind v2020, EIGN achieves a 12% reduction in RMSE and a 6% improvement in *R*_p_ compared to GIGN on CASF-2016.

### Ablation study

To evaluate the effectiveness of each component in our model, we created five variants of EIGN:

A. Replacing NAE with MLP.
B. Removing edge update mechanism.
C. Removing edge perturbation layer.
D. Using only GINE to handle intra- and inter-molecular interactions.
E. Applying the gating mechanism from NAE to subsequent modules.

**Fig 4:**
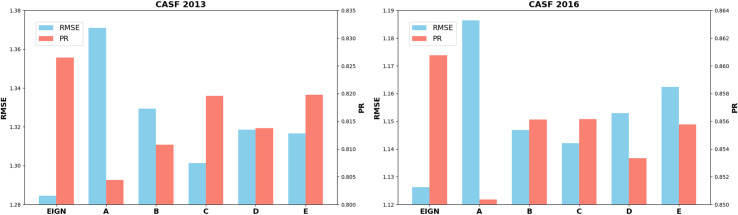
Prediction performance of EIGN and four variants on CASF-2013 and CASF-2016.

We trained these variant models and assessed their predictive performance on CASF-2013 and CASF-2016, enabling us to analyze the contribution of each component to the overall model performance. A comparison of the prediction results for the different variants on two datasets is shown in Fig 4.

From Fig 4, it can be observed that replacing NAE with MLP has the greatest impact on the performance of the model. This modification significantly increased the RMSE and decreased *R*_p_, indicating that NAE plays a crucial role in capturing node features and the global information of the graph. The other modules contribute supplementarily to edge feature updates, structural noise handling, and local information representation. Maintaining these modules helps to improve the generalization ability and stability of the model in chemical structure data.

### Performance evaluation on the non-PDBbind external test Set

**Fig 5:**
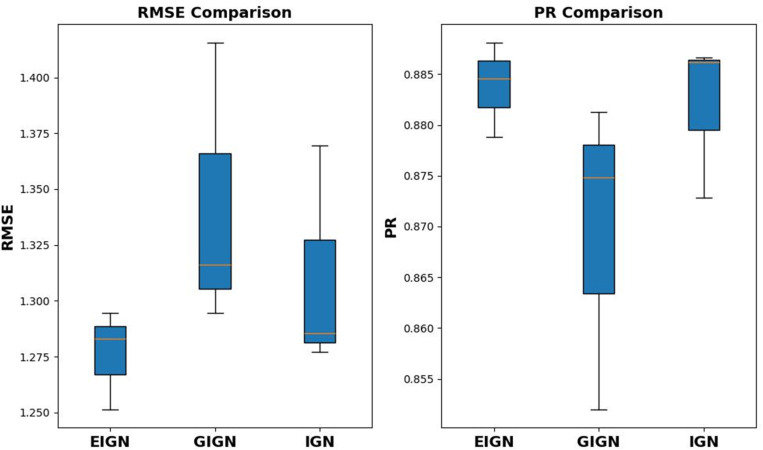
Comparison of prediction performance of EIGN, GIGN, and IGN on the CSAR-HIQ-set.

To validate the generalization capability of EIGN and its predictive performance on non-PDBbind datasets, we used the CSAR-NRC dataset as an external test set for performance evaluation. Using the same training set and strategy, we trained two interaction GNNs, GIGN and IGN, and compared their prediction performance on the CSAR-NRC dataset with that of our model. As shown in Fig 5, EIGN outperforms GIGN and IGN on the CSAR-HIQ-set, demonstrating higher prediction accuracy and good generalization ability.

### Analysis of node feature contribution

To further analyze the contribution of different node features to the performance of EIGN, we conducted ablation studies and feature importance analysis.

**Fig 6:**
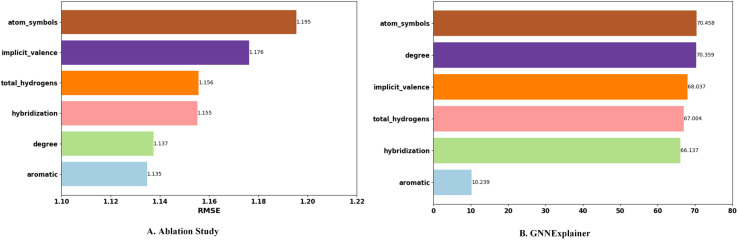
Contribution analysis of the node feature components in the model. (A) Ablation study. (B) GNNExplainer.

In the ablation study, we sequentially removed different node features, retrained the model, and evaluated its performance on the test set to observe the impact of each feature. The experimental results are shown in Fig 6A. The removal of atomic symbols and implicit valence features has a significant impact on model performance, indicating that these features play a crucial role in the prediction task.

Additionally, we used GNNExplainer [52] to analyze the importance of node features. The procedure involved loading the trained best model, interpreting it on specific samples, and generating a feature importance map. This map illustrates the contribution of each feature in the model. The explanation results are shown in Fig 6B. Except for aromatic features, there was no significant difference in the importance of various feature groups, suggesting that aromatic features have a minor influence on model performance.

From Fig 6, it can be observed that aromatic features consistently have the smallest impact on model performance, which indicates that aromatic information contributes little to the EIGN. However, the impact of removing atomic degree features in the ablation study was minor, which differs from the results in GNNExplainer. This discrepancy may be due to our model already adopting degree-related edge features, hence the removal of degree features did not result in a significant decline in model performance.

### Impact of dataset similarity on prediction results

We analyzed the impact of dataset similarity on prediction results. Specifically, we evaluated the structural similarity between proteins in the PDBbind v2020 refined set and those in CASF2016. Similarity was quantified using the TM-score [53], a widely used metric for structural alignment. Samples with a TM-score greater than 0.5 were considered similar.

**Fig 7:**
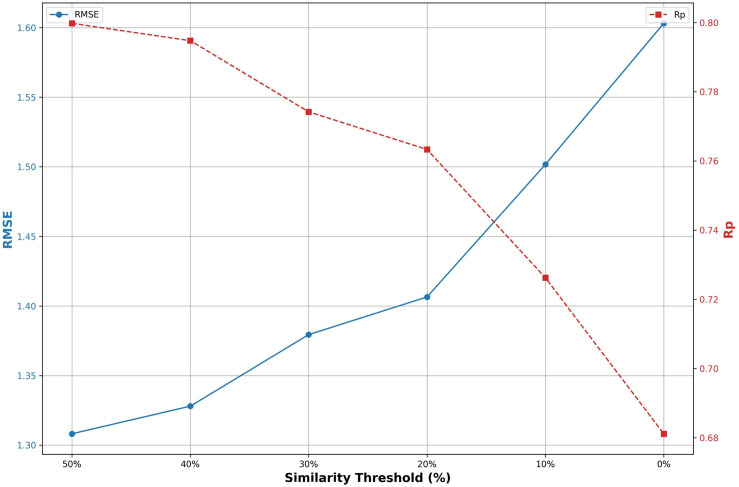
The impact of the proportion of similar samples in the training set on model prediction performance.

Based on this criterion, after removing samples that overlap with CASF2016, we obtained 1,734 similar samples and 3,236 remaining samples from the PDBbind v2020 refined set. Using these samples, we constructed six training sets, each containing 3,000 samples, with the proportions of similar samples set at 0%, 10%, 20%, 30%, 40%, and 50%. We trained EIGN using these training sets and evaluated its prediction performance on CASF2016. As shown in Fig 7, the model achieves better performance on the test set when the training set contains a higher proportion of similar samples. For example, when the proportion of similar samples increases from 0% to 50%, the prediction accuracy of the model rises substantially. This observation suggests that incorporating a more diverse range of protein structures in the training data is crucial for achieving more accurate predictions. Therefore, future data collection efforts should prioritize covering a variety of protein structures, especially those currently underrepresented or missing in the training set. This approach will enhance the generalization performance and predictive capability of the model.

### Performance of the model in virtual screening tasks

After training the model on the PDBbind dataset, we further evaluated its virtual screening capabilities. Specifically, we focused on the PYGM target from the DUD-E dataset and conducted ligand docking analyses using both experimental structures and those predicted by AlphaFold3 [54]. To assess the reliability of the predicted structure, we performed a global structural alignment with TM-align [55]. The alignment produced a TM-score of 0.711—well above the 0.5 threshold—confirming that both structures share the same fold. We then concentrated on the active pocket region, defined as the 108 residues located within 5Å of the ligand in the experimental structure, and observed a local Root Mean Square Deviation (RMSD) of 0.47Å. Based on these validation results, we carried out restricted molecular docking with AutoDock Vina, setting the search space as a 20Å cube centered on the active pocket.

**Fig 8:**
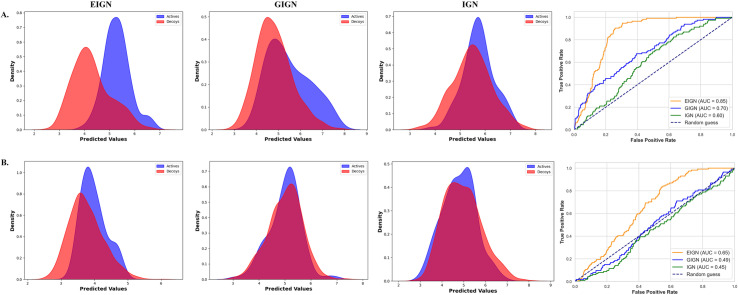
Visualization of the performance of EIGN, GIGN, and IGN on the PYGM target. (A) Experimentally resolved protein structure. (B) Alphafold3-predicted protein structure.

To evaluate the prediction results, we conducted a visual analysis of the outcomes for both experimental setups. Density plots and ROC curves were generated, as shown in Fig 8.

Analysis of the density plots for the experimentally resolved protein structure indicates that the EIGN model excels in distinguishing active compounds from inactive ones. The ROC curve analysis further supports this, with the EIGN model achieving an AUC value of 0.85, outperforming the other two models.

For the AlphaFold3-predicted protein target, although the EIGN model still surpassed the other models, there was a noticeable decline in its overall performance. This may be attributed to the precision limitations of the AlphaFold3 predicted structures and deviations in the active site region.

### Prediction ability of the model for ligands with undetermined structures

To evaluate the prediction ability of the model for ligands with undetermined structures, we conducted experimental analysis on the Cyclin-Dependent Kinase 2 (CDK2) target from the ChEMBL database. The structure of each ligand was generated using the RDKit tool based on the Simplified Molecular Input Line Entry System (SMILES) representation of the ligands.

**Fig 9:**
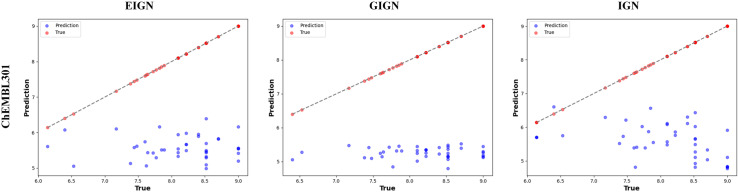
Scatter plots of the prediction results of EIGN, GIGN, and IGN on the CDK2 target.

As shown in Fig 9, the prediction results of EIGN, GIGN, and IGN for this target were visualized in scatter plots. The results indicate that for target CDK2, the predictions from EIGN, GIGN, and IGN were suboptimal, as the predicted affinity values showed minimal variation across multiple ligands for the same target.

The poor performance of the three models on the CDK2 target may be closely related to the characteristics of the PDBbind dataset. The PDBbind dataset consists of protein-ligand complexes, where each protein is typically associated with a single high-resolution ligand. This structure trains models to recognize the specific features of a protein interacting with a single high-affinity ligand, rather than handling scenarios where a protein is associated with multiple ligands. In contrast, the ChEMBL dataset is more diverse; a single target is often associated with numerous ligands, exhibiting a broader range of binding affinities. Additionally, the ligands for the CDK2 target display a high degree of similarity, which might make it challenging for the model to differentiate subtle variations among these ligands.

Although the dataset characteristics largely determine the performance of the model, the model itself may also have limitations. EIGN primarily extracts graph structural features through the GNN layers when processing protein-ligand complexes. In cases where multiple potential ligands exist for the same target, the model struggles to distinguish subtle structural differences or variations in affinity between ligands. For example, the message passing and feature aggregation in GNN layers tend to smooth node features, reducing sensitivity to local structural differences in ligands.

Future work could address these limitations by incorporating multi-source training, such as integrating data from ChEMBL or other multi-ligand target datasets during model training. Additionally, the ability of the model to capture ligand differences could be enhanced by introducing additional constraints or attention mechanisms to better focus on ligand similarities, such as molecular fingerprints or topological features.

## Conclusion

In this study, we proposed an innovative GNN-based model for PLA prediction, and it is named EIGN. This model captures inter- and intra-molecular interactions through a multi-level message-passing mechanism within GCN. Our model integrates inter- and intra-molecular message-passing strategies to better capture diverse interaction patterns. Additionally, we incorporate an edge update mechanism to enhance the representational capacity of edge features, enabling a more comprehensive modeling of relationships within the graph.

To verify the performance of the model, we performed evaluations on high-quality datasets such as CASF-2013, CASF-2016, and CSAR-NRC set. Based on the evaluation indicators RMSE and *R*_p_, the experimental results show that EIGN exhibits significant performance advantages in PLA prediction.

Despite the significant performance improvements, the model still has potential for further enhancement, particularly in screening capabilities. Future research should focus on optimizing this aspect to augment its practical applicability.
